# Pain or gain?

**DOI:** 10.7554/eLife.73378

**Published:** 2021-09-29

**Authors:** Valeria Kalienkova

**Affiliations:** University of Groningen Groningen Netherlands

**Keywords:** TMEM120, coenzyme A, membrane protein, mechanosensation, ion channel, cryo-EM structure, Human, Mouse

## Abstract

The 3D structures of a membrane protein called TMEM120A suggest that it may act as an enzyme in fat metabolism rather than as an ion channel that senses mechanical pain.

**Related research article** Rong Y, Jiang J, Gao Y, Guo J, Song D, Liu W, Zhang M, Zhao Y, Xiao B, Liu Z. 2021. TMEM120A contains a specific coenzyme A-binding site and might not mediate poking- or stretch-induced channel activities in cells. *eLife* **10**:e71474. doi: 10.7554/eLife.71474

All cells are surrounded by a lipid membrane that is not permeable to ions and other solutes. To enter or leave a cell, therefore, ions and solutes must pass through proteins that are embedded in the lipid membrane. This gatekeeping role means that transmembrane proteins are involved in a number of physiological processes, and if they fail to work properly, the result can be a severe metabolic defect.

Membrane proteins belonging to the TMEM120 family have been associated with a wide variety of functions, and recently it was reported that one family member – TMEM120A (also referred to as TACAN) – was highly expressed in clusters of neurons called dorsal root ganglia, and was involved in the sensing of mechanical pain (Beaulieu-Laroche et al., 2020). This study suggested that TMEM120A was a channel protein that, when activated by a mechanical stimulus, allowed positive ions to cross the lipid membrane by passing through a pore within the protein. Now, in eLife, Zhenfeng Liu, Bailong Xiao, Yan Zhao and colleagues at the Chinese Academy of Sciences and Tsinghua University report the results of a series of cryo-electron microscopy and electrophysiology experiments that reveal new details about this protein and call its role in the detection of mechanical pain into question (Rong et al., 2021).

First, the researchers – who include Yao Rong, Jinghui Jiang and Jianli Guo as joint first authors – used cryo-electron microscopy to determine the three-dimensional structure of TMEM120A. This revealed a rather novel architecture: the protein is made up of two monomers, both of which contain six segments that have a helical shape (Figure 1). It is possible that ions can pass through a channel formed by the six transmembrane helices in each monomer. However, the 3D structure also revealed the presence of a molecule bound to the inside of the putative channel that would prevent the passage of ions through it. Rong et al. hypothesized that this molecule was an endogenous ligand that had remained tightly bound to the protein when it was being purified prior to the cryo-electron microscopy experiments, and then used mass spectrometry to show that the molecule was coenzyme A (CoASH). This coenzyme is primarily known for helping to synthesize and degrade various cellular compounds, including fatty acids (Gout, 2018), and it had not previously been associated with mechanosensation.

**Figure 1.**
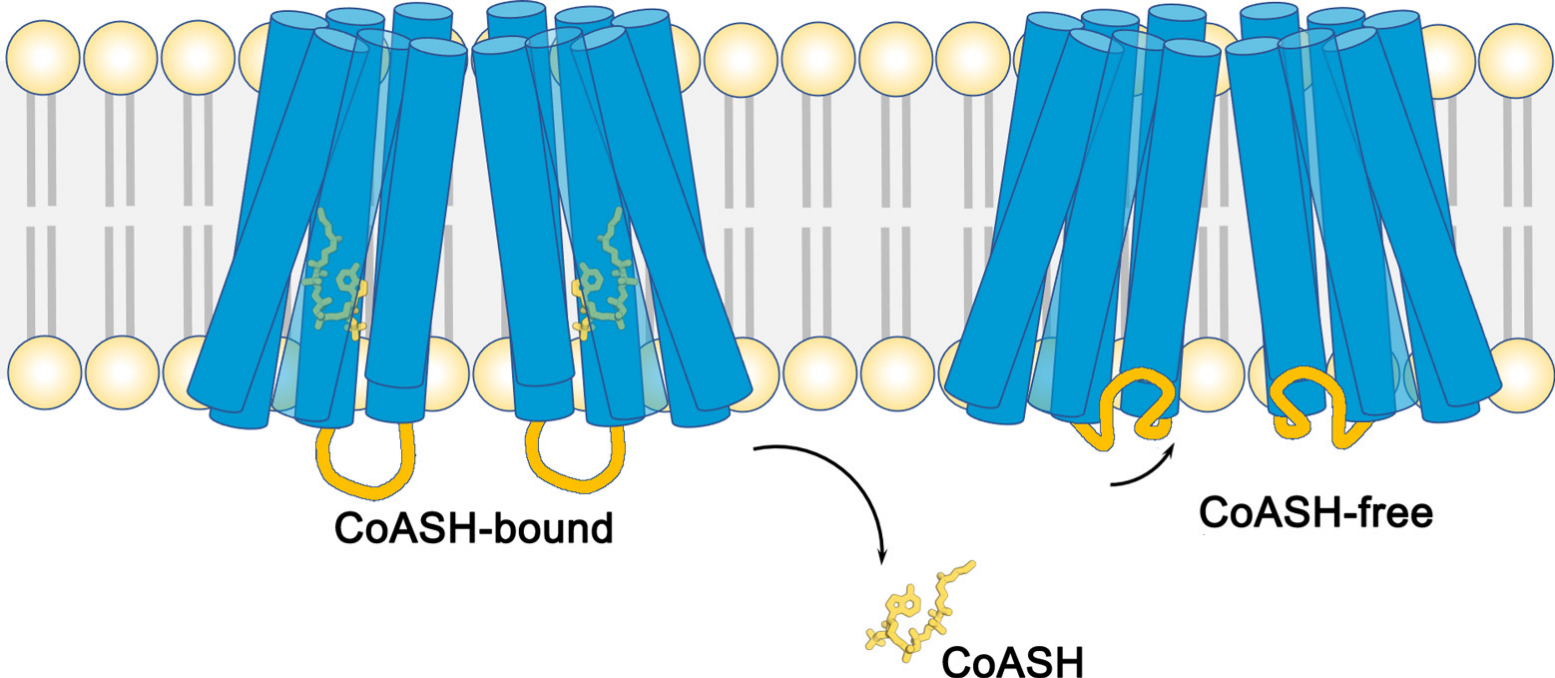
The structure of the membrane protein TMEM120A. Schematic showing the structures of the membrane-embedded region of the protein TMEM120A. This region of the protein is comprised of two monomers, both of which contain six helical segments (blue cylinders) that span across the lipid bilayer (pale yellow and gray). When a coenzyme called CoASH (yellow) is present, it binds to a site inside the helical segments (left). When CoASH is not present (right), two of the cytoplasmic loops (orange) that connect helical segments change their position to cover the entrance to the binding site. Cytoplasmic loops that do not change their position are not shown.

To investigate this further, Rong et al. purified TMEM120A without the coenzyme and determined its 3D structure. The two structures were similar, apart from the shape of two cytoplasmic loops: in the CoASH-bound state the loops extended into the cytoplasm of the cell; however, when CoASH was not present the loops partially closed the entrance to the channel in each monomer (Figure 1). Rong et al. then used these structures to identify which amino acid residues are involved in the binding of CoASH. Further experiments showed that mutating one of these residues, a conserved tryptophan, dramatically decreased the affinity of TMEM120A for CoASH.

By conclusively showing that TMEM120A specifically interacts with CoASH, the work of Rong et al. suggests that this membrane protein might in fact work as a membrane-embedded enzyme. It is also notable that Rong et al., along with several other groups (Ke et al., 2021; Niu et al., 2021; Parpaite et al., 2021; Rong et al., 2021; Xue et al., 2021), could not reproduce the mechanosensitive currents reported previously. Moreover, there are several lines of evidence to suggest that TMEM120A has a greater role in lipid metabolism. Originally, TMEM120A was shown to be important for fat tissue differentiation (Batrakou et al., 2015). Several recent reports have shown that *C. elegans* and mammalian cells need it to accumulate fat, and that disrupting the gene for TMEM120A leads to metabolic defects in mice fed a high-fat diet (Czapiewski et al., 2021; Li et al., 2021). In addition, its structure also resembles that of ELOVL7, a membrane-embedded enzyme that helps to elongate fatty acids (Nie et al., 2020; Niu et al., 2021; Xue et al., 2021). However, TMEM120A does not catalyze the same reactions as ELOVL7, and the possible substrates and end products of TMEM120A remain unknown (Niu et al., 2021).

These findings do not completely rule out a role for TMEM120A as a mechanosensitive channel. Computational modeling suggests that it might be able to act as an ion channel in the absence of CoASH (Chen et al., 2021). Alternatively, the addition of extra subunits may be required for it to work as an ion channel (Chen et al., 2021). Furthermore, TMEM120A may contribute to mechanosensation by modulating other mechanosensitive channels in dorsal root ganglia (Del Rosario et al., 2021). Undoubtedly, the work by Rong et al. provides new insights into proteins belonging to the TMEM120 family, and the controversy surrounding their role will likely fuel future scientific endeavors.

## References

[bib1] Batrakou DG, de Las Heras JI, Czapiewski R, Mouras R, Schirmer EC (2015). TMEM120A and B: Nuclear envelope transmembrane proteins important for adipocyte differentiation. PLOS ONE.

[bib2] Beaulieu-Laroche L, Christin M, Donoghue A, Agosti F, Yousefpour N, Petitjean H, Davidova A, Stanton C, Khan U, Dietz C, Faure E, Fatima T, MacPherson A, Mouchbahani-Constance S, Bisson DG, Haglund L, Ouellet JA, Stone LS, Samson J, Smith MJ, Ask K, Ribeiro-da-Silva A, Blunck R, Poole K, Bourinet E, Sharif-Naeini R (2020). TACAN is an ion channel involved in sensing mechanical pain. Cell.

[bib3] Chen X, Wang Y, Li Y, Lu X, Chen J, Li M, Wen T, Liu N, Chang S, Zhang X, Yang X, Shen Y (2021). Cryo-EM structure of the human TACAN channel in a closed state. bioRxiv.

[bib4] Czapiewski R, Batrakou DG, de las Heras J, Carter RN, Sivakumar A, Sliwinska M, Dixon CR, Webb S, Lattanzi G, Morton NM, Schirmer EC (2021). Genomic loci mispositioning in *Tmem120a* knockout mice yields latent lipodystrophy. bioRxiv.

[bib5] Del Rosario JS, Gabrielle M, Yudin Y, Rohacs T (2021). TMEM120A/TACAN inhibits mechanically activated Piezo2 channels. bioRxiv.

[bib6] Gout I (2018). Coenzyme A, protein CoAlation and redox regulation in mammalian cells. Biochemical Society Transactions.

[bib7] Ke M, Yu Y, Zhao C, Lai S, Su Q, Yuan W, Yang L, Deng D, Wu K, Zeng W, Geng J, Wu J, Yan Z (2021). Cryo-EM structures of human TMEM120A and TMEM120B. bioRxiv.

[bib8] Li Y, Huang S, Li X, Yang X, Xu N, Qu J, Mak HY (2021). The endoplasmic reticulum-resident protein TMEM-120/TMEM120A promotes fat storage in *C. elegans*. bioRxiv.

[bib9] Nie L, Pike ACW, Pascoa TC, Bushell SR, Quigley A, Ruda GF, Chu A, Cole V, Speedman D, Moreira T, Shrestha L, Mukhopadhyay SMM, Burgess-Brown NA, Love JD, Brennan PE, Carpenter EP (2020). The structural basis of fatty acid elongation by the ELOVL elongases. bioRxiv.

[bib10] Niu Y, Tao X, Vaisey G, Olinares PDB, Alwaseem H, Chait BT, MacKinnon R (2021). Analysis of the mechanosensor channel functionality of TACAN. eLife.

[bib11] Parpaite T, Brosse L, Séjourné N, Laur A, Mechioukhi Y, Delmas P (2021). Patch-seq of mouse DRG neurons reveals candidate genes for specific mechanosensory functions. bioRxiv.

[bib12] Rong Y, Jiang J, Gao Y, Guo J, Song D, Liu W, Zhang M, Zhao Y, Xiao B, Liu Z (2021). TMEM120A contains a specific coenzyme A-binding site and might not mediate poking- or stretch-induced channel activities in cells. eLife.

[bib13] Xue J, Han Y, Baniasadi H, Zeng W, Pei J, Grishin NV, Wang J, Tu BP, Jiang Y (2021). TMEM120A is a coenzyme A-binding membrane protein with structural similarities to ELOVL fatty acid elongase. eLife.

